# CD97/*ADGRE5* in Cancer: Structural Activation, Context-Dependent Signaling, and Therapeutic Targeting

**DOI:** 10.3390/cells15171605

**Published:** 2026-09-03

**Authors:** Yuhong Lei, Yuan Zhang, Yufeng Wang, Lingyu Li

**Affiliations:** 1 The First Bethune Hospital of Jilin University, Changchun 130021, China; 2 Institute of Cancer, Jilin University, Changchun 130021, China; 3 Medical Publishing Center, The First Bethune Hospital of Jilin University, Changchun 130021, China; 4 International Center of Future Science, Jilin University, Changchun 130021, China

**Keywords:** CD97, *ADGRE5*, adhesion G-protein-coupled receptor, structural activation, cancer, targeted therapy

## Abstract

**Highlights:**

**What are the main findings?**

- CD97 is involved in tumor stemness, invasion, metastasis, and cell survival through adhesion-related signaling, mechanosensing, and downstream pathway activation.
- CD97-targeted chimeric antigen receptor (CAR) therapies have shown antitumor activity in animal models, whereas CD97-targeted antibody–drug conjugates (ADCs) are currently supported by in vitro proof-of-concept evidence and RNA-mediated downregulation remains at an early exploratory stage.

**What are the implications of the main findings?**

- High CD97 expression does not necessarily mean that a tumor will respond to CD97-targeted treatment.
- Patient selection should consider tumor dependence on CD97, normal-tissue expression, and potential on-target/off-tumor toxicity.

**Abstract:**

CD97 is an adhesion G-protein-coupled receptor encoded by *ADGRE5* that integrates extracellular signals (including cell adhesion, ligand binding, and mechanical stimulation) with intracellular signal transduction. Recent structural studies have further elucidated tethered/intramolecular agonist (TIA)/Stachel recognition and engagement of the seven-transmembrane domain (7TMD), activation-associated 7TMD conformational changes, and G-protein coupling, including the structural basis for the preferential coupling of CD97 to G13. Currently, antibody–drug conjugates (ADCs) targeting CD97 are supported by in vitro proof-of-concept evidence, whereas chimeric antigen receptor (CAR) strategies have shown antitumor activity in animal models of glioblastoma (GBM) and acute myeloid leukemia (AML). Existing research indicates that CD97 is involved in maintaining stem-like states, invasion and metastasis, metabolic adaptation, and stress survival in certain tumors, and its function varies depending on tumor type and cellular environment. Because CD97 is also expressed in normal immune cells and various nonhematopoietic tissues, systemic targeted therapy may be limited by on-target/off-tumor toxicity. This article reviews the latest advances in CD97 structure and signal transduction, and explores its tumor-related functions, biomarker value, evidence for ADC and CAR-related therapies, as well as early exploratory directions involving RNA-mediated downregulation and structure-guided interventions.

## 1. Introduction

The initiation, progression, metastasis and treatment response of cancer not only depend on the intrinsic characteristics of tumor cells, but are also dynamically influenced by immune cells, stromal cells, the extracellular matrix and soluble factors in the tumor microenvironment [1,2,3]. These microenvironment components affect the plasticity, invasion and metastasis of tumor cells through cell-to-cell contact, cell–matrix interaction, mechanical stimulation and paracrine signaling. Therefore, membrane receptors that can sense changes in cell contact and the extracellular environment and convert them into intracellular signals may constitute an important functional interface connecting tumor cells with the tumor microenvironment.

Adhesion G-protein-coupled receptors (aGPCRs) belong to this type of receptor. According to the current terminology of the International Union of Basic and Clinical Pharmacology (IUPHAR) and the Adhesion GPCR Consortium, aGPCRs are composed of the extracellular N terminus (ENT), a seven-transmembrane domain (7TMD), and the intracellular C terminus (ICT) in terms of topological structure; ENT usually contains domains involved in ligand recognition and cell adhesion. Many aGPCRs are believed to respond to extracellular inputs such as ligand binding, cell contact or mechanical stimulation, and generate context-dependent signal outputs through G-protein-dependent mechanisms and other mechanisms [4,5,6]. CD97 is one of the earliest studied members of this family and also one of the earliest members associated with cancer.

In 1994, Eichler et al. used the monoclonal antibody BL-Ac(F2) to first identify CD97 as a surface antigen associated with the early activation of lymphocytes. This antigen was not detected in resting lymphocytes, but its level increased rapidly after T cells and B cells were activated; in contrast, myeloid-monocytic cells showed constitutive expression of the antigen, indicating that this activation-dependent property was mainly applicable to lymphocytes rather than all leukocyte populations [7]. This antigen was subsequently named CD97; in 1995, Hamann et al. cloned its cDNA and confirmed that it encodes a receptor with N-terminal epidermal growth factor (EGF)-like domains and a C-terminal seven-transmembrane region [8]. Subsequently, according to the current IUPHAR nomenclature, CD97 was classified into the EGF-TM7/aGPCR family, and its encoding gene was named adhesion G-protein-coupled receptor E5 (*ADGRE5*) [6]. CD97 is upregulated in acute myeloid leukemia (AML), glioblastoma (GBM), and their stem-cell compartments, and antibodies targeting the GPCR autoproteolysis-inducing (GAIN) domain have been developed as antibody–drug conjugates (ADCs) and evaluated in vitro [9]. In 1997, Aust et al. first systematically reported the high expression of CD97 in dedifferentiated thyroid carcinomas and its correlation with lymph node involvement, establishing an early link between CD97 and tumor dedifferentiation and disease progression [10]. In this review, “CD97” refers to the receptor protein, and “*ADGRE5*” refers to its encoding gene. As an aGPCR, CD97 has a characteristic modular structure, in which extracellular recognition, receptor processing, and 7TMD activation jointly constitute the structural basis for downstream signal transduction.

The ENT of CD97 contains a variable number of EGF-like repeats and a GAIN domain harboring a GPCR proteolysis site (GPS). Autoproteolysis at the GPS generates an N-terminal fragment (NTF) and a C-terminal fragment (CTF), which usually remain associated as a noncovalent NTF–CTF complex. The N terminus of the CTF contains a tethered/intramolecular agonist (TIA; also known as Stachel). When its accessibility increases, this sequence can interact intramolecularly with the orthosteric pocket of the 7TMD and promote receptor activation, providing a structural basis for converting extracellular inputs such as ligand binding, cell contact, and mechanical stimulation into intracellular signals [6,11,12].

A 2022 review by Aust et al. summarized the existing roles of CD97 in tumor cell adhesion, migration, and metastasis [13]. Post-review studies have expanded our understanding of CD97 structure activation and context-dependent signaling. Cryo-electron microscopy (cryo-EM) studies have revealed the differences between CD97 inactive and active states, TIA/Stachel recognition mechanisms, and the structural basis for preferential coupling to G13 [11,12]. Cancer research has also linked CD97 to GBM stemness and metabolic reprogramming, and found its association with inflammation-driven epithelial–mesenchymal transition (EMT) and invasion in other cancers [14,15,16]. Meanwhile, the physiological roles of CD97 in immune cell mechanosensing, tissue localization, and stabilizing dendritic cell (DC)–T cell immune synapses have been further elucidated [17,18]. These findings collectively indicate that CD97 signaling is highly context-dependent and is influenced by receptor state, extracellular input, cell type, and local environment.

These mechanistic advances have also driven therapeutic exploration of CD97 targeting strategies. Recent studies have provided in vitro proof-of-concept evidence for a CD97-targeted ADC in AML and GBM cells and patient-derived glioblastoma stem-like cells (GSCs).

Chimeric antigen receptor (CAR)-Th9 cells and CD97-directed CAR-T cells optimized by CD97 knockout have shown antitumor activity in GBM and AML animal models, respectively [9,14,19]. However, CD97 is also expressed in normal immune cells and various nonhematopoietic tissues, raising potential on-target/off-tumor toxicity concerns and indicating that tumor antigen levels alone are insufficient to determine the feasibility of treatment. This review therefore focuses on CD97 structural activation, context-dependent signaling, and cancer-associated functions; compares the levels of evidence supporting ADCs, CAR-based approaches, and RNA-mediated downregulation; and discusses patient stratification, therapeutic response, and platform-specific safety considerations.

## 2. Structural Basis and Activation Mechanisms of CD97

### 2.1. Extracellular N Terminus: EGF-like Repeats, Splice Variants, and Ligand Recognition

**Figure 1:**
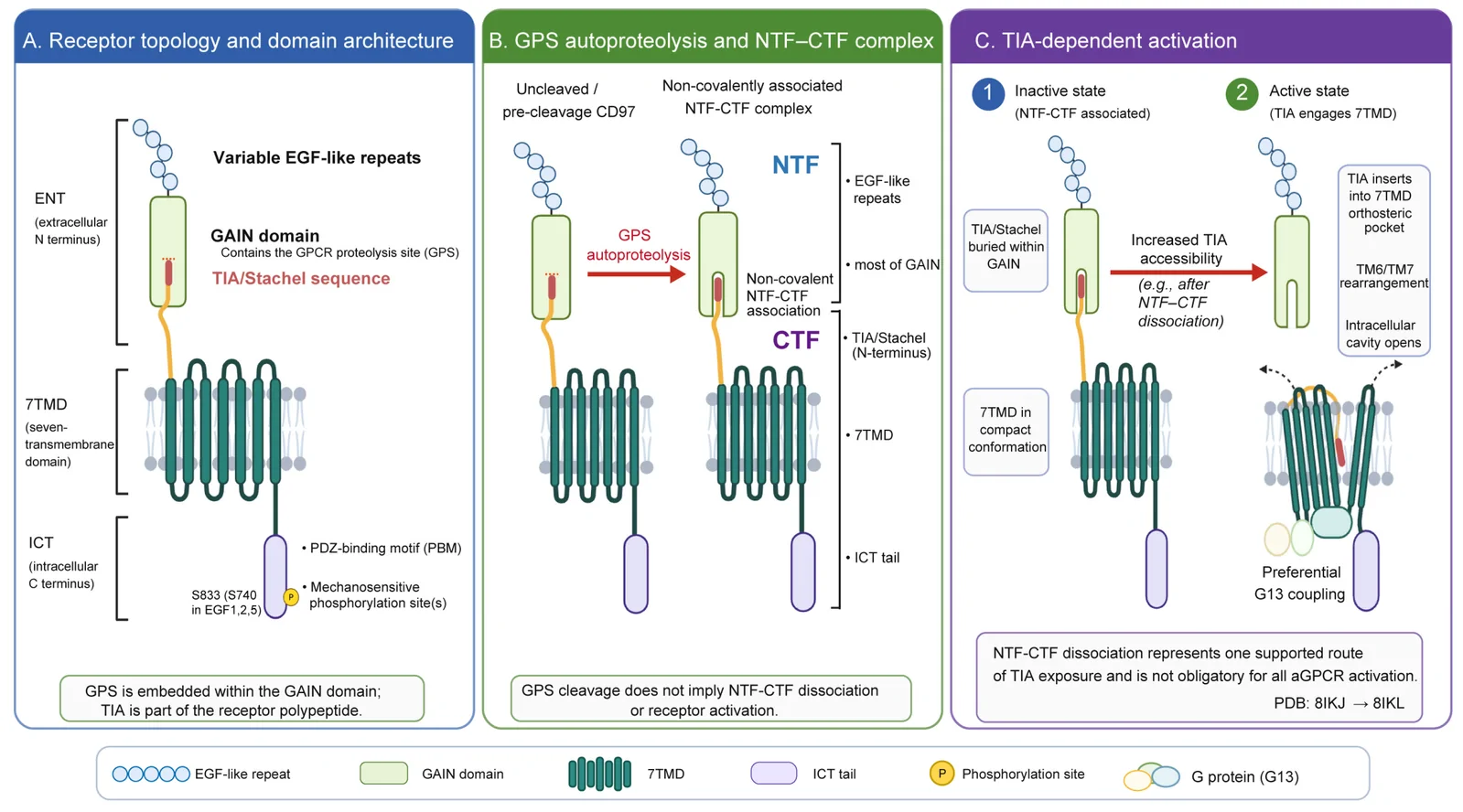
Structural features, autoproteolytic processing, and TIA-dependent activation of CD97. (**A**) CD97 consists of epidermal growth factor (EGF)-like repeats, a GPCR autoproteolysis-inducing (GAIN) domain, a seven-transmembrane domain (7TMD), and an intracellular C-terminus (ICT). The GPCR proteolysis site (GPS) is located within the GAIN domain. The tethered/intramolecular agonist (TIA, i.e., the Stachel sequence) is located at the N terminus of the C-terminal fragment (CTF). The ICT contains a C-terminal PDZ-binding motif (PBM) with a mechanically regulated serine phosphorylation site. This site corresponds to S833 in CD97(EGF1–5) and S740 in CD97(EGF1,2,5). (**B**) Autoproteolytic cleavage of CD97 at the GPS generates an N-terminal fragment (NTF) and a membrane-embedded CTF. The NTF contains the EGF-like repeats and most of the GAIN domain, whereas the CTF contains the TIA, 7TMD, and ICT. After cleavage, the NTF and CTF can remain associated through noncovalent interactions. Therefore, GPS cleavage itself does not imply NTF–CTF dissociation, nor is it equivalent to receptor activation. (**C**) In the inactive state, the TIA is buried within the GAIN domain, restricting its access to the binding pocket of the 7TMD. When TIA accessibility increases, for example after NTF–CTF dissociation exposes it, the TIA can fold back into the orthosteric binding pocket of the 7TMD. TIA binding induces pronounced bending and rearrangement of transmembrane helices 6 and 7 (TM6 and TM7). These changes open an intracellular G-protein-binding cavity that accommodates Gα13, consistent with the preferential coupling of CD97 to G13. Current evidence supports NTF–CTF dissociation as one route to TIA exposure, but this process is not universally required for adhesion GPCR activation. Notably, NTF–CTF dissociation was not directly captured in the 8IKJ/8IKL structural comparison. The inactive apo CD97 GAIN–7TMD construct and the G_13_-bound active CD97 CTF construct correspond to PDB entries 8IKJ and 8IKL, respectively. Neither structure includes the EGF-like repeats or the distal ICT containing the PBM. Arrows indicate cleavage or conformational transitions, and colors distinguish the structural domains and components as defined in the figure key. This figure is for illustrative purposes only and is not drawn to scale. Abbreviations: CTF, C-terminal fragment; EGF, epidermal growth factor; ENT, extracellular N-terminus; GAIN, GPCR autoproteolysis-inducing domain; GPCR, G-protein-coupled receptor; GPS, GPCR proteolysis site; ICT, intracellular C-terminus; NTF, N-terminal fragment; PBM, PDZ-binding motif; PDB, Protein Data Bank; TIA, tethered/intramolecular agonist; TM, transmembrane helix; 7TMD, seven-transmembrane domain. Created in BioRender. Lei, Y. (2026) https://BioRender.com/vpvmiwy.

From distal to membrane-proximal, the ENT of CD97 contains a variable number of EGF-like repeats followed by a GAIN domain (Figure 1A). The extracellular diversity of CD97 arises mainly from alternative splicing of EGF-repeat exons, which generates protein variants containing three, four, or five EGF-like repeats [20,21]. The common CD97(EGF1,2,5) variant has relatively high affinity for CD55, whereas larger variants containing the fourth EGF-like repeat can also bind glycosaminoglycans such as dermatan sulfate (also known as chondroitin sulfate B) [22,23,24].

The EGF-like repeats form the principal extracellular ligand-recognition platform of CD97. CD97 and CD55 form a calcium-dependent, low-affinity, antiparallel complex with rapid binding kinetics, properties suited to transient adhesion and mechanical signal transmission during cell contact and under shear [23,25]. Alternative splicing therefore changes not only extracellular architecture but also ligand selectivity and binding behavior, generating distinct microenvironment-dependent inputs.

Glycosylation also affects ENT structure and function. N-glycosylation within the EGF-like repeats influences CD55 binding, antibody-epitope accessibility, and CD97 detection, whereas site-specific N-glycosylation can regulate GPS autoproteolysis [26,27]. In contrast, O-glycosylation of CD97 remains poorly characterized. Large-scale O-glycoproteomic analyses have reported candidate O-linked glycosylation sites in CD97, including Thr265 [28], but these observations have not been validated in CD97-focused biochemical studies. Their site occupancy, glycan structures, cell-type dependence, and functional consequences therefore remain unknown.

### 2.2. Membrane-Proximal GAIN/GPS: Autoproteolysis and the NTF–CTF Complex

The membrane-proximal GAIN domain comprises subdomains A and B; in CD97, subdomain A is relatively short and lacks several α-helical elements found in other aGPCRs. The more structurally conserved, β-strand-rich subdomain B contains the GPS, immediately followed by the terminal β-strand that constitutes the GAIN-bound TIA/Stachel sequence. The GPS is not an independent domain, but the autoproteolytic cleavage site within the GAIN domain (Figure 1A,B). CD97 was among the first aGPCRs shown to undergo autoproteolytic cleavage at this site. Cleavage at the GPS generates an NTF and a CTF. The NTF comprises the EGF-like repeats and most of the GAIN domain. The CTF begins with the final β-strand of the GAIN domain, which constitutes the GAIN-bound TIA/Stachel segment. It also contains the GAIN–7TMD linker, the complete 7TMD, and the ICT. Following cleavage, the NTF and CTF generally remain noncovalently associated as an NTF–CTF complex (Figure 1B) [6,29,30].

Autoproteolytic cleavage at the GPS, NTF–CTF dissociation, and receptor activation are not the same event (Figure 1B). After cleavage at the GPS, NTF and CTF can still maintain binding through noncovalent interactions as an NTF–CTF complex; therefore, cleavage alone does not indicate that the NTF–CTF complex has dissociated or that the receptor has been activated. This processing step can also be influenced by N-glycosylation and receptor structural context [26,31]. In addition to canonical autoproteolytic cleavage at the GPS, other proteolytic cleavage events can also regulate CD97. For example, the lysine-specific gingipain (Kgp) of *Porphyromonas gingivalis* can cleave CD97 after Lys290 within the ENT and increase Tango reporter activity [32]. These findings indicate that CD97 proteolytic processing is not a single pathway and that its effect on subsequent activation depends on the specific cleavage event and receptor context.

### 2.3. TIA/7TMD: Release of Autoinhibition, Conformational Activation, and G-Protein Coupling

The TIA (also known as the Stachel sequence) is located at the N-terminus of the CTF and is part of the receptor polypeptide, rather than an independent extracellular ligand. In the cleaved but nondissociated NTF–CTF complex, the TIA forms the C-terminal β-strand (S14) of GAIN subdomain B and is embedded within the GAIN domain, thus restricting its access to the orthosteric binding pocket of the 7TMD (Figure 1C).

Recent cryo-EM studies have further elucidated the conformational differences between the inactive and active states of CD97. In the inactive apo CD97 structure (Protein Data Bank (PDB) ID: 8IKJ), the 7TMD is in a compact conformation, with the extracellular ends of transmembrane helices 6 and 7 (TMH6 and TMH7) shifting inward to form a restricted orthosteric binding pocket. The contacts between the GAIN domain and extracellular loops 1 and 2 (ECL1 and ECL2), as well as the hydrophobic interactions within the triad tethering motif (W545–Y683–F760), collectively stabilize the autoinhibited state [11].

In the dissociation-dependent TIA activation model, NTF–CTF dissociation enhances the accessibility of the TIA. Subsequently, the N-terminal segment of the TIA transitions from a β-strand to a partially α-helical conformation and inserts into the orthosteric binding pocket of the 7TMD (Figure 1C; PDB ID: 8IKL). This transition expands both the extracellular orthosteric pocket and the intracellular G-protein-binding cavity and rotates the conserved toggle-switch residue W6.53, thereby promoting the formation of the receptor’s active conformation [6,11].

The activity of the CD97 TIA/Stachel sequence is residue-dependent. In an engineered CD97 C-terminal-fragment assay, alanine or lysine substitutions at F533 and L536 markedly impaired TIA-dependent reporter activity, whereas several other positions were more tolerant to substitution; for example, I535K retained approximately 40% of wild-type activity [33]. Structural studies show that F533, I535, L536, and M537 contribute to hydrophobic contacts that stabilize the TIA within the orthosteric binding pocket of the 7TMD [12]. These findings indicate that Stachel sequence composition can influence CD97 activation efficiency. However, whether naturally occurring or cancer-associated variants in this region alter CD97 signaling has not yet been established.

The structures of CD97 complexes with G13, Gq, and Gs further clarify the structural basis of G-protein-coupling preference. Compared with Gαq and Gαs, the α5 helix of Gα13 inserts deeper into the intracellular G-protein-binding cavity of CD97 and forms closer contacts with TMH3, TMH5, and TMH6, providing a structural explanation for CD97’s preferential coupling to G13 (Figure 1C) [12]. However, G13 is unlikely to be the only G-protein-coupling partner of CD97.

The available CD97 structures support a model in which NTF–CTF dissociation promotes TIA exposure, but this mechanism does not apply to all aGPCRs. Some receptors can undergo dissociation-independent TIA-dependent activation while the NTF–CTF complex remains intact; furthermore, TIA-independent signaling has been described [6,31]. Whether CD97 employs other activation mechanisms under endogenous expression and physiological stimulation remains unclear.

### 2.4. Intracellular C Terminus: Mechanotransduction and Scaffold Protein Regulation

On the intracellular side, CD97 signaling can be further regulated by G proteins, β-arrestin recruitment, and ICT-binding partners. The antiparallel configuration of the CD97–CD55 complex provides a structural basis for bearing intercellular mechanical forces [25]. In splenic conventional type 2 dendritic cells (cDC2s), erythrocyte CD55 can promote the release of the CD97 NTF from the NTF–CTF complex in a force-dependent manner under shear stress and trigger Gα13-dependent migratory and transcriptional responses [17]. Complementary in vitro work showed that mechanical stimulation generated by orbital shaking promotes CD97-dependent β-arrestin-2 recruitment, whereas CD55 neutralization markedly attenuates this response [31]. These findings indicate that CD55-dependent mechanical inputs can alter the state of the extracellular CD97 fragment and transmit these mechanical inputs to intracellular signaling pathways.

The distal ICT of CD97 contains a PDZ-binding motif (PBM) that interacts with the scaffold protein discs large homolog 1 (DLG1). Mechanical stimulation induces phosphorylation of a key serine residue within this motif, weakens the CD97–DLG1 interaction, and alters actin cytoskeleton organization, cellular mechanical properties, and cell detachment under shear stress [34]. Therefore, the response of CD97 to mechanical stimulation involves not only receptor processing and 7TMD signaling but also ICT phosphorylation and scaffold protein reorganization, with the latter two processes further affecting cell behavior.

Overall, extracellular-to-intracellular regulatory layers shape context-dependent CD97 signaling: ENT splice variants, glycosylation, and the ligand environment influence extracellular input; GPS processing and the state of NTF–CTF association regulate TIA accessibility; the TIA sequence and 7TMD conformation influence receptor activation and G-protein coupling; and ICT phosphorylation and scaffold interactions regulate intracellular output. These structural determinants and their functional and therapeutic implications are summarized in Table 1.

## 3. Context-Dependent Signaling and Cancer-Associated Functions of CD97

CD97 functions in cancer in a highly context-dependent manner. Its effects depend on G proteins, β-arrestins, and other pathways that can participate in signal transduction within tumor cells, and are also influenced by membrane receptor complexes, extracellular ligands, and the local microenvironment. CD97 can also extend its influence to the tumor microenvironment by acting on surrounding cells through heterotypic cell contact, extracellular fragments, or extracellular vesicles [6]. The following sections will discuss cell-autonomous signaling, contextual input and membrane complexes, and transcellular or non-cell-autonomous outputs.

### 3.1. Cell-Autonomous Signaling Configurations: G Proteins, Beta-Arrestin, and Tumor Cell State

CD97 can influence tumor cell behavior through multiple intracellular pathways. Heterologous expression systems have shown that CD97 can functionally couple with multiple Gα subunits, including Gα12/13, Gα14, and Gαz [36]; recent structural studies have further shown that it preferentially couples to G13. In prostate cancer cells, CD97 promotes invasion through Gα12/13-Rho-related signaling [37]. In addition to classical G-protein coupling, studies in different tumor contexts have also found that CD97 is associated with the mechanistic target of rapamycin complex 2 (mTORC2)–protein kinase B (AKT), β-arrestin–mitogen-activated protein kinase (MAPK), and Janus kinase 2 (JAK2)/signal transducer and activator of transcription 3 (STAT3) pathways, indicating that its intracellular output varies with cell type and state.

**Figure 2:**
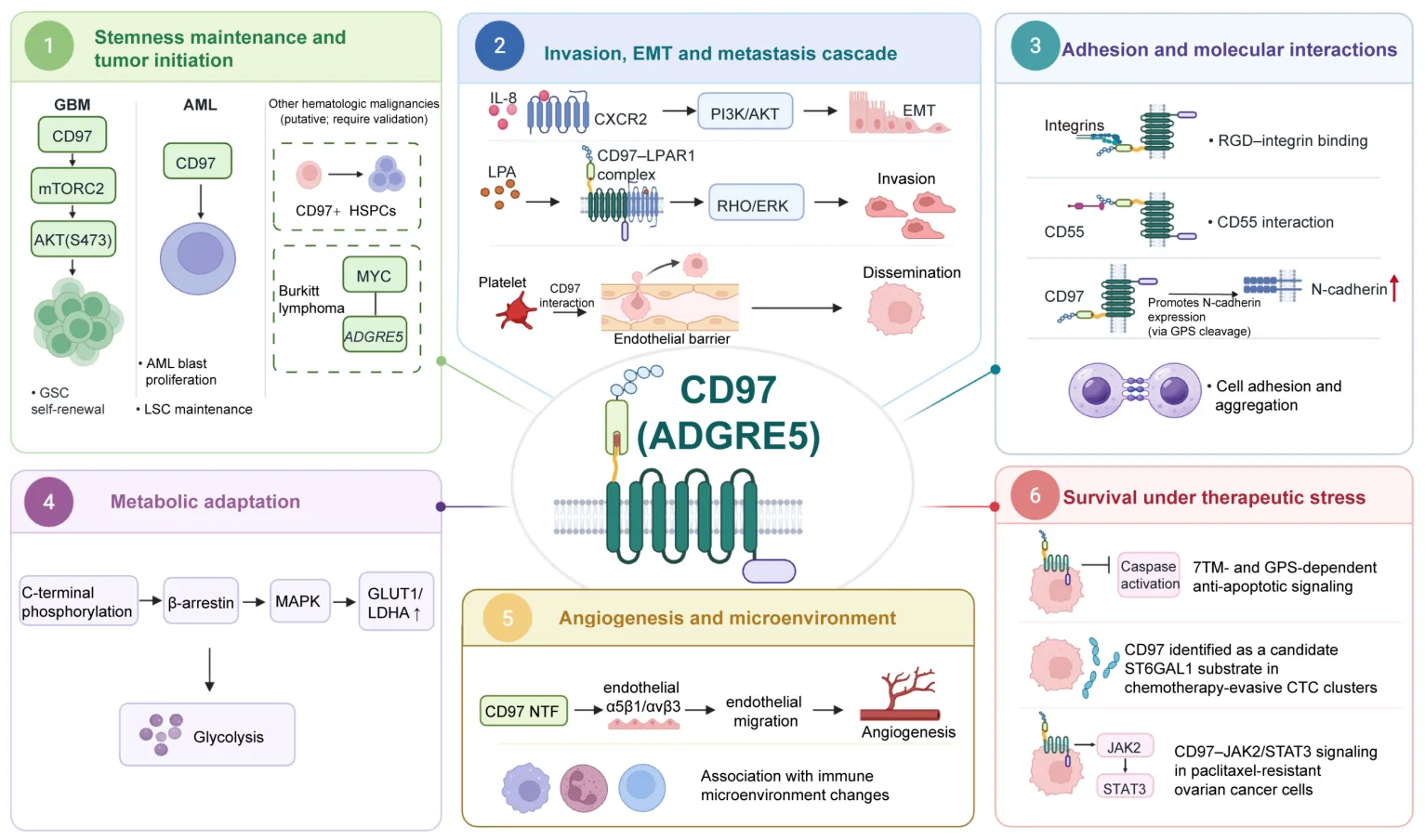
Context-dependent roles of CD97 in cancer. CD97 has been linked to stemness and tumor initiation, invasion and metastasis, adhesion, metabolic adaptation, angiogenesis, microenvironmental changes, and stress-related survival in selected tumor models. These effects involve context-specific signaling and interactions with extracellular ligands, adhesion molecules, and membrane receptors. GPS cleavage can promote N-cadherin expression and homotypic aggregation, but direct CD97–N-cadherin binding has not been demonstrated. CD97 has also been identified as a candidate ST6GAL1 substrate in chemotherapy-evasive CTC clusters, although a direct causal role in chemoresistance remains unclear. Colors are used solely to distinguish the six functional modules. Arrows indicate the direction of reported signaling, regulatory, or cellular processes, whereas blunt-ended lines indicate inhibitory effects. The diagram is schematic and does not imply that all mechanisms operate across all tumor types. *ADGRE5*, adhesion G-protein-coupled receptor E5; AKT, protein kinase B; AML, acute myeloid leukemia; CTC, circulating tumor cell; CXCR2, C-X-C motif chemokine receptor 2; EMT, epithelial–mesenchymal transition; ERK, extracellular signal-regulated kinase; GBM, glioblastoma; GLUT1, glucose transporter 1; GPS, GPCR proteolysis site; GSC, glioblastoma stem-like cell; HSPC, hematopoietic stem/progenitor cell; IL-8, interleukin-8; JAK2, Janus kinase 2; LDHA, lactate dehydrogenase A; LPA, lysophosphatidic acid; LPAR1, lysophosphatidic acid receptor 1; LSC, leukemia stem cell; MAPK, mitogen-activated protein kinase; mTORC2, mechanistic target of rapamycin complex 2; NTF, N-terminal fragment; PI3K, phosphoinositide 3-kinase; RGD, arginine–glycine–aspartate; ST6GAL1, β-galactoside α-2,6-sialyltransferase 1; STAT3, signal transducer and activator of transcription 3; 7TMD, seven-transmembrane domain. Created in BioRender. Lei, Y. (2026) https://BioRender.com/hiquu3r.

GBM provides one of the most mechanistically developed examples (Figure 2, panel 1). In patient-derived GSCs, CD97 supports self-renewal and tumorigenicity through mTORC2-AKT Ser473 signaling; CD97 knockdown reduces GSC proliferation, stem-like properties, and in vivo tumor formation, whereas mTORC2 inhibition partially attenuates these phenotypes [14,15]. In the same tumor context, phosphorylation of the CD97 ICT promotes β-arrestin recruitment and MAPK signaling and increases expression of glycolysis-related proteins such as glucose transporter 1 (GLUT1) and lactate dehydrogenase A (LDHA), thereby supporting glycolytic metabolism and tumor initiation (Figure 2, panel 4) [15]. Thus, even within a single tumor type, CD97 can generate distinct functional outputs according to the intracellular signaling environment.

Similar context dependence is evident in other models. In AML, CD97 supports proliferation and survival of leukemic blasts and helps maintain an immature state and leukemia stem-cell function [38]. Related expression associations have been reported in *JAK2/CALR*-mutant myeloproliferative neoplasm stem/progenitor cells and *MYC*-associated Burkitt lymphoma [39,40]. In HT1080 cells, CD97 overexpression reduces cell death induced by several proapoptotic stimuli, whereas CD97 knockdown or disruption of the 7TMD increases caspase-dependent apoptosis (Figure 2, panel 6) [41]. CD97 has also been associated with JAK2/STAT3 signaling in metastatic or paclitaxel-resistant ovarian cancer cells [42]. In addition, glycoproteomic analysis identified CD97 as a potential β-galactoside α-2,6-sialyltransferase 1 (ST6GAL1) substrate in chemoresistant circulating tumor cell clusters, but current evidence does not establish that hyposialylation of CD97 itself directly determines paclitaxel resistance [43]. Overall, available data support context-specific roles for CD97 in stemness, metabolic adaptation, and stress survival rather than a universal role as a driver of drug resistance.

### 3.2. Contextual Inputs and Membrane Complexes: Lipid Mediators, Adhesion Partners, and Inflammatory Signals

CD97 output is also shaped by local extracellular inputs and membrane-complex composition. CD97 can form a complex with lysophosphatidic acid receptor 1 (LPAR1), enhance lysophosphatidic acid (LPA)-dependent Rho and extracellular signal-regulated kinase (ERK) signaling, and promote invasion and disease progression in prostate and thyroid cancer models (Figure 2, panel 2) [37,44]. CD55 is the best-characterized extracellular ligand of CD97; it binds the EGF-like repeats in the ENT and participates in cell-contact and mechanical signaling [23,25,45]. Integrin α5β1/αvβ3 and CD90 on activated endothelial cells have also been reported to bind or functionally interact with CD97 [46,47]. An Arg-Gly-Asp (RGD) motif in CD97 also contributes to integrin-related adhesion and viability in HT1080 cells [48]. These interactions are not equivalent: LPAR1 is primarily supported as a functional receptor complex, CD55 has relatively direct extracellular ligand evidence, and integrins and CD90 are supported by different types of binding and functional experiments.

The relationship between CD97 and cadherins is better understood as adherens-junction localization and downstream expression control than as established direct receptor–ligand binding (Figure 2, panel 3). In normal intestinal epithelium, CD97 localizes to E-cadherin-mediated lateral adherens junctions and is associated with β-catenin; during colorectal tumorigenesis, this junctional localization and CD97–β-catenin association are progressively lost, and CD97 becomes more intracellular [49,50]. This shift suggests that the subcellular localization of CD97 itself can be remodeled during tumor progression. In HT1080 cells, GPS autoproteolysis of CD97 promotes N-cadherin expression and enhances N-cadherin-dependent homotypic aggregation, but there is no evidence that CD97 binds N-cadherin directly [51]. Thus, E-cadherin-related studies primarily reflect the localization and complex association of CD97 at adherens junctions, whereas N-cadherin represents downstream CD97-dependent expression control.

Inflammatory factors can also alter CD97 output. In intrahepatic cholangiocarcinoma, interleukin-8 (IL-8) upregulates CD97 through C-X-C motif chemokine receptor 2 (CXCR2)–phosphoinositide 3-kinase (PI3K)/AKT signaling, and this process is accompanied by EMT-related changes, including reduced E-cadherin and increased N-cadherin and vimentin; knockdown or neutralization of CD97 attenuates IL-8-induced tumor cell invasion [16]. These findings indicate that local inflammatory and adhesive environments can shape CD97 signaling by changing its expression, membrane-complex composition, and cellular adhesion state.

### 3.3. Transcellular and Non-Cell-Autonomous Outputs: Vasculature, Platelets, Extracellular Vesicles, and the Immune Microenvironment

Whereas the preceding section considered how extracellular factors regulate CD97, this section focuses on how CD97 influences nearby and distant cells through heterotypic contact, extracellular fragments, or extracellular vesicles. These effects include actions of CD97-expressing cells on other cell types and bidirectional signals that depend on heterotypic cell contact, even when some terminal responses occur within the tumor cell itself.

In the vascular compartment, purified CD97 NTF binds endothelial α5β1 integrin, with αvβ3 also contributing to adhesion, and promotes endothelial migration and angiogenesis in vivo (Figure 2, panel 5) [46]. CD97-expressing cells can additionally enhance angiogenesis through RGD-related and N-cadherin–matrix metalloproteinase-9 (MMP-9) mechanisms [52]. These findings indicate that both the extracellular region of CD97 and CD97-associated cellular signaling can affect endothelial behavior, but they do not establish constitutive widespread NTF shedding in tumors.

Interactions between CD97 and platelets are most relevant during hematogenous dissemination and extravasation. Tumor-cell-surface CD97 can directly contact platelets and trigger bidirectional signaling that increases tumor cell invasiveness, disrupts endothelial-junction integrity, and promotes transendothelial migration [53]. Prostate cancer models further show that transcellular receptor–ligand pairs such as CD55–CD97 and αIIbβ3-CD97 promote calcium mobilization, invasion, and resistance to apoptosis [54]. Although some terminal effects occur in tumor cells, their induction depends on transcellular input supplied by platelets.

CD97-associated effects can also extend to distant tissues through extracellular vesicles. In gastric cancer, exosomes derived from CD97-high tumor cells enhance MAPK-related signaling in recipient cells and promote proliferation and invasion [55]. Exosomes from highly metastatic cells also increase tumor cell accumulation and the expression of premetastatic-niche-associated molecules in draining lymph nodes, whereas CD97 knockdown attenuates these effects [56]. The relative contribution of CD97 protein itself versus other CD97-associated exosomal cargo cannot yet be fully distinguished; these findings are therefore best described as “CD97-associated extracellular-vesicle signaling.”

Evidence linking CD97 to the tumor immune microenvironment remains comparatively limited. In GBM, transcriptomic deconvolution associated high CD97 expression with increased signatures of unpolarized (M0) macrophages, regulatory T cells, and resting natural killer cells, and coculture experiments suggested that CD97 expression promotes differentiation of THP-1 human monocytic leukemia cells toward macrophages [57]. Compared with studies involving endothelial cells, platelets, and extracellular vesicles, a direct mechanistic role for CD97 in remodeling the immune microenvironment requires further validation.

Overall, CD97 can be viewed as a context-dependent signaling-integration node whose functions are jointly shaped by the intracellular signaling environment of tumor cells, local extracellular inputs, and transcellular interactions. Depending on context, CD97 contributes to several interconnected functional modules, including stemness, cell adhesion and invasion, metastasis, angiogenesis, metabolic adaptation, intercellular communication, and cell survival under stress (Figure 2), although the strength of evidence differs among mechanisms. Studies have implicated CD97 in anti-apoptotic effects and the maintenance of stem-like states, which may support tumor cell survival under nutrient deprivation, proapoptotic stimuli, and therapeutic stress. Therefore, CD97 is more appropriately viewed as a factor that promotes cell survival and adaptation under therapeutic stress rather than as an established driver of drug resistance. CD97 also has important physiological functions in normal immune cells and is expressed in several nonhematopoietic tissues, which should be considered when evaluating potential on-target/off-tumor toxicity and the therapeutic window of CD97-targeted therapy.

## 4. CD97 Expression in Normal Tissues and the Safety Basis for Targeted Therapy

CD97 is not a tumor-specific antigen. In addition to its broad expression across leukocyte populations, including granulocytes, monocytes/macrophages, and DCs [58], CD97 is expressed by several normal nonhematopoietic cell types. A recent comprehensive analysis of adhesion GPCRs found CD97 predominantly in immune cells but also detectable in smooth muscle, skeletal muscle, and selected epithelial cells; earlier histological studies similarly demonstrated CD97 expression in smooth muscle of the urinary bladder, bronchi, myometrium, and gastrointestinal tract [6,59]. In normal intestinal epithelium, CD97 also localizes to E-cadherin-associated lateral adherens junctions [49]. Potential on-target/off-tumor toxicity during systemic CD97 targeting may therefore involve both immune and nonhematopoietic tissues.

CD97 expression in normal immune cells has established physiological significance. In the spleen, sensing of erythrocyte CD55 by CD97 regulates the positioning and function of cDC2s and marginal-zone B cells [17,60]. The CD97–CD55 axis also contributes to granulocyte homeostasis, recruitment to inflammatory sites, and antibacterial host defense [61,62]. CD97 on DCs stabilizes DC–T cell immunological synapses and supports subsequent T cell activation [18]. Thus, CD97 in normal tissues not only constitutes a potential antigen reservoir but also participates in physiological tissue positioning, cell contact, and immune regulation.

Existing studies provide several direct safety warnings. Under acute inflammatory conditions, some anti-CD97 antibodies can cause granulocytopenia or granulocyte depletion through fragment crystallizable (Fc) receptor-dependent mechanisms [63]. Antibody treatment can also delay neutrophil migration and impair antibacterial host defense, an effect not reproduced by *Adgre5* gene targeting [64,65]. In CD97-targeted CAR-T studies, CD97 expression by the therapeutic T cells themselves causes fratricide, which can be reduced by CD97 knockout [19]. By contrast, systematic safety data for smooth muscle, epithelial cells, and other nonhematopoietic tissues remain very limited. The therapeutic window of CD97 therefore cannot be judged solely from expression differences between tumors and peripheral blood mononuclear cells (PBMCs); it must also account for antigen abundance in normal tissues, potential on-target/off-tumor toxicity, the therapeutic epitope, the distribution of drug exposure, and the mechanism of the specific platform.

## 5. Biomarker Value and Patient Stratification

CD97/*ADGRE5* is currently better regarded as a candidate marker for risk assessment and therapeutic stratification than as a stand-alone clinical decision marker. In several solid tumors, CD97 or CD97–CD55 expression has been associated with invasive-front localization, advanced stage, lymph node metastasis, vascular or lymphatic invasion, and poor survival, including colorectal, gastric, gallbladder, pancreatic, and cervical cancers [66,67,68,69,70,71,72]. High CD97 expression in GBM is likewise associated with invasiveness and poor prognosis, and functional dependence on CD97 has been demonstrated in patient-derived models [15,73,74,75]. Most clinical studies, however, are retrospective and differ in sample size, antibody choice, assay platform, and scoring criteria; current evidence is therefore insufficient to establish CD97 as an independent prognostic marker.

Soluble CD97 has also been explored for disease monitoring. In patients with intrahepatic cholangiocarcinoma, soluble CD97 in bile, together with tissue CD97/CD55 expression, was associated with lymph node metastasis and poor prognosis [76]. CD97 in pleural effusions and in the serum of patients receiving chemotherapy has also been examined in preliminary studies [77,78]. These findings suggest potential monitoring value for body-fluid CD97 but require standardized assays and validation in independent cohorts.

Evidence in hematological malignancies is most concentrated in AML. High CD97 expression is associated with poor overall survival and features such as FMS-like tyrosine kinase 3 internal tandem duplication (*FLT3*-ITD) [79,80,81], and CD97 has been incorporated into multiparameter flow-cytometry panels to distinguish leukemic from normal hematopoietic cells and support measurable residual disease monitoring [82]. Its practical value is more likely to arise in combination with other leukemia-associated markers than from CD97 alone.

These clinical associations do not establish predictive value for therapy. For CD97-targeted treatment, the more important task is to identify patients whose tumors show sufficient functional dependence and adequate tumor–normal antigen separation. Single-cell and transcriptomic studies indicate that the significance of *ADGRE5* depends strongly on its cellular source. CD97-associated T cell states have been linked to antitumor immunity and immune-checkpoint-inhibitor response [83,84,85], and *ADGRE5*-related signals to metastasis-associated tumor cell states and inferred tumor–immune communication [86,87]; these findings remain largely correlative.

**Figure 3:**
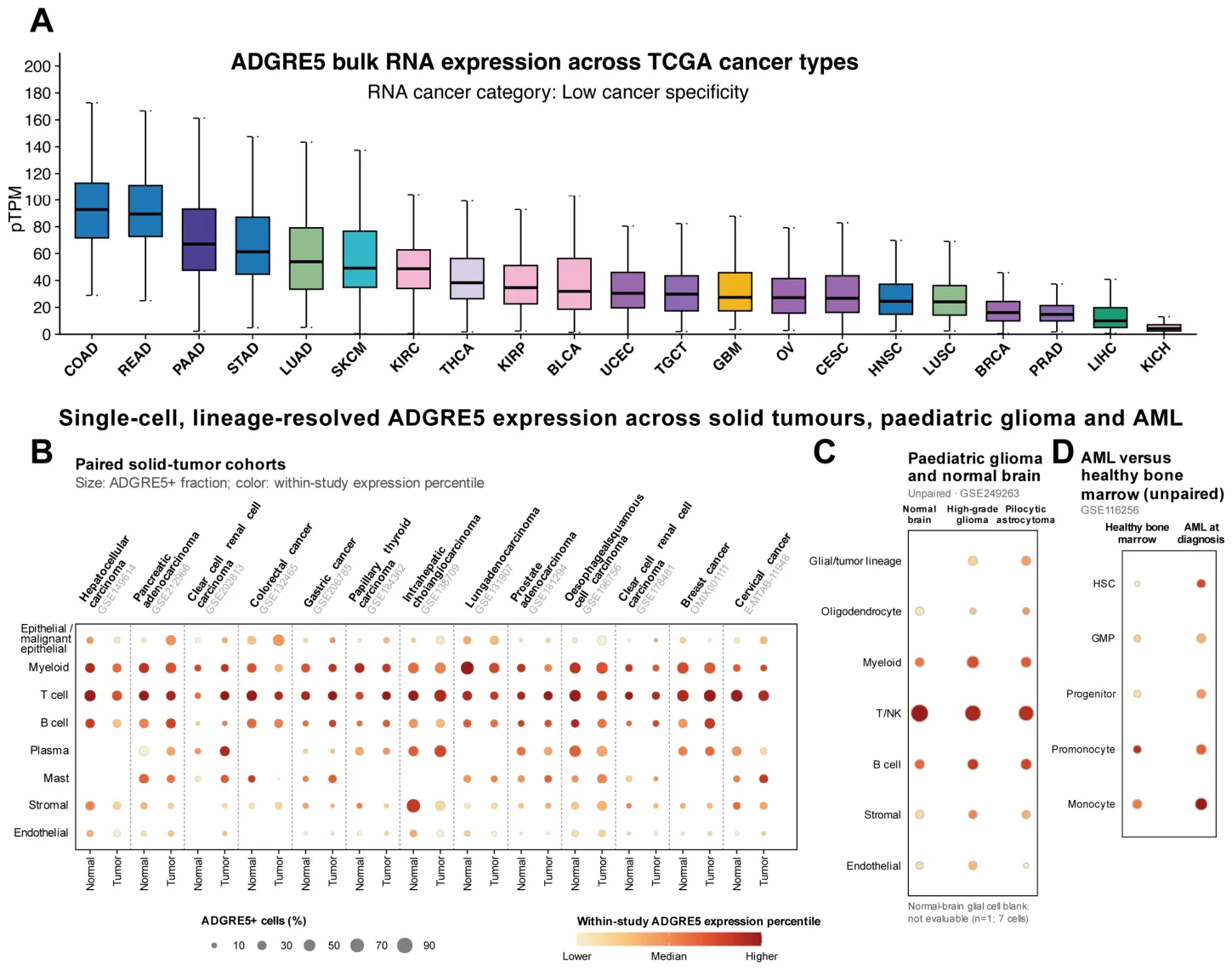
Bulk and lineage-resolved *ADGRE5* expression across human cancers. (**A**) *ADGRE5* bulk RNA expression across TCGA cancer types. RNA-sequencing data were obtained from the Human Protein Atlas and are shown as protein-coding transcripts per million (pTPM). Bulk-tissue measurements reflect the combined contribution of malignant, immune, stromal and other cell populations and therefore do not resolve the cellular source of *ADGRE5* expression. (**B**) Bubble plots show the detection and relative expression levels of *ADGRE5* in different cell lineages of paired normal and tumor tissues across 13 solid-tumor cohorts. Epithelial cells in tumor tissues were classified as malignant only when explicitly annotated as malignant by the authors, or when aneuploidy was inferred using CopyKAT with patient-matched normal cells as a diploid reference. The area of the circle represents the percentage of *ADGRE5*-positive cells. The color indicates the percentile rank of median *ADGRE5* expression at the patient level within the study, calculated in each cohort based on both normal and tumor samples, as well as all presented cell lineages. Therefore, colors can be used to compare relative expression between different cell populations and conditions within the same dataset, but do not represent directly comparable absolute expression levels between different datasets. (**C**) Detection and relative expression levels of *ADGRE5* in normal brain tissue, high-grade glioma (HGG), and pilocytic astrocytoma (PA) samples from GSE249263. The area of the circle represents the median proportion of *ADGRE5*-positive cells at the patient level, and the color indicates the percentile ranking of median *ADGRE5* expression within the study. Due to the limited cell count, glial/tumor cell lineages in normal brain tissue could not be reliably assessed; therefore, this section is left blank. The samples in each group were not patient-paired. (**D**) Detection and relative expression levels of *ADGRE5* in different hematopoietic cell populations in healthy bone marrow and newly diagnosed acute myeloid leukemia (AML) in GSE116256. The area of the circle represents the median proportion of *ADGRE5*-positive cells at the donor level, and the color indicates the percentile ranking of median *ADGRE5* expression within the study. Healthy bone marrow and AML samples were not paired. Gray or blank entries indicate that data were unavailable or could not be reliably assessed. AML, acute myeloid leukemia; GMP, granulocyte–monocyte progenitor; HGG, high-grade glioma; HSC, hematopoietic stem cell; NK, natural killer; PA, pilocytic astrocytoma. Cancer-type abbreviations: BLCA, bladder urothelial carcinoma; BRCA, breast invasive carcinoma; CESC, cervical squamous cell carcinoma and endocervical adenocarcinoma; COAD, colon adenocarcinoma; GBM, glioblastoma multiforme; HNSC, head and neck squamous cell carcinoma; KICH, kidney chromophobe; KIRC, kidney renal clear cell carcinoma; KIRP, kidney renal papillary cell carcinoma; LIHC, liver hepatocellular carcinoma; LUAD, lung adenocarcinoma; LUSC, lung squamous cell carcinoma; OV, ovarian serous cystadenocarcinoma; PAAD, pancreatic adenocarcinoma; PRAD, prostate adenocarcinoma; READ, rectum adenocarcinoma; SKCM, skin cutaneous melanoma; STAD, stomach adenocarcinoma; TGCT, testicular germ cell tumors; THCA, thyroid carcinoma; UCEC, uterine corpus endometrial carcinoma.

Our exploratory analyses of The Cancer Genome Atlas (TCGA) bulk RNA-sequencing data and publicly available single-cell RNA-sequencing datasets further show marked variation in *ADGRE5* expression across cancer types, cellular lineages, and normal–tumor conditions (Figure 3; Table S1; Data S1; Data S2). The single-cell analyses provide lineage-resolved comparisons within individual datasets, but the resulting relative expression measures are not directly comparable across datasets and do not represent absolute cell-surface antigen abundance or directly define a therapeutic window. Companion diagnostics for CD97-targeted therapy should therefore address not only whether CD97 is highly expressed, but also which cells express it, whether it is present at the cell surface, whether the tumor depends on CD97, and whether sufficient antigen separation exists between the tumor and critical normal tissues. Surface antigen density, epitope accessibility, and receptor internalization must then be evaluated in a platform-specific manner.

## 6. CD97-Targeted Therapy: Platform-Specific Evidence and Therapeutic Windows

**Figure 4:**
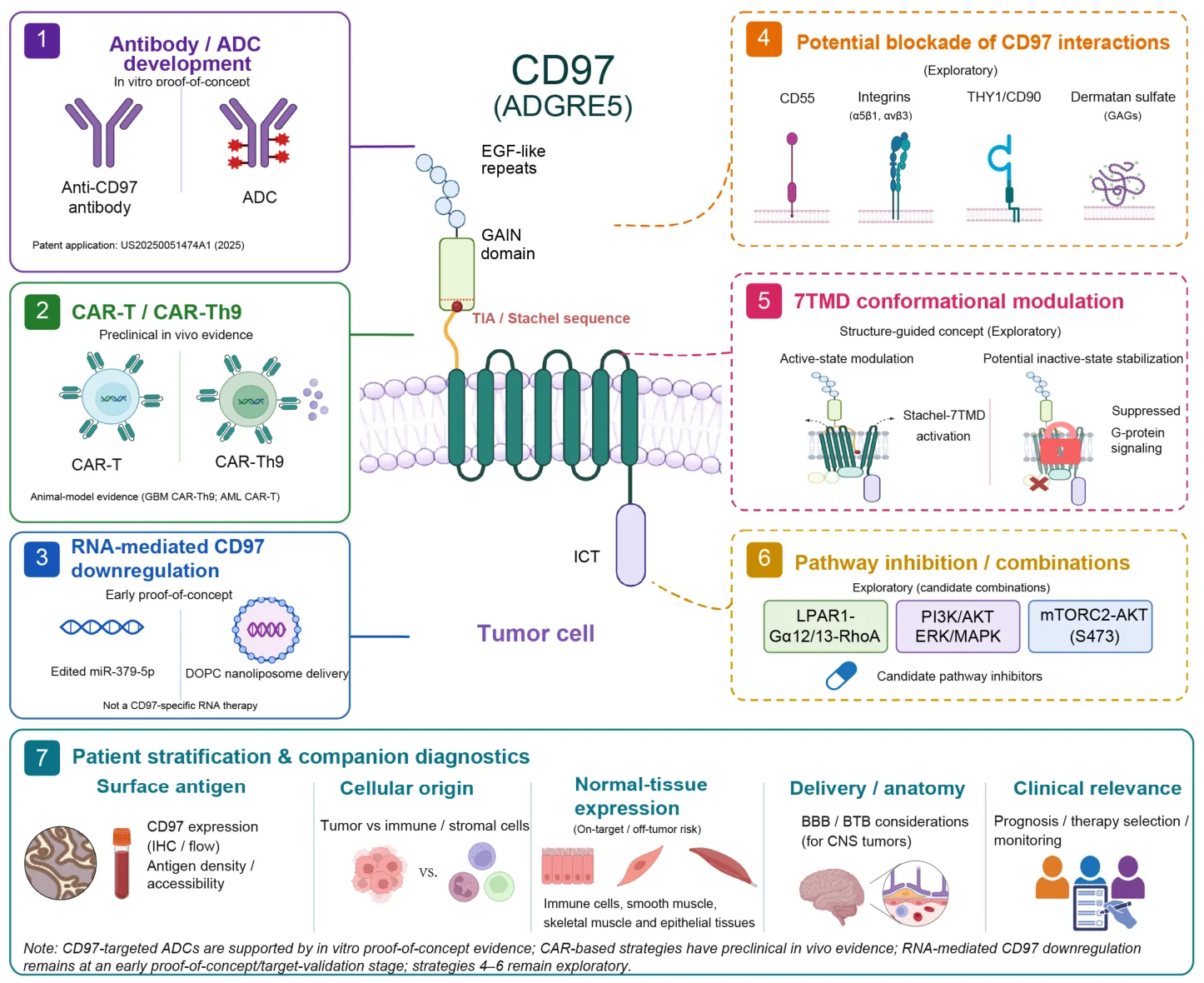
Therapeutic and translational framework for targeting CD97. Current and proposed strategies include antibody/ADC development, CAR-based cell therapies, RNA-mediated CD97 downregulation, blockade of extracellular interactions, 7TMD conformational modulation, downstream pathway inhibition, and patient stratification. The level of evidence differs across platforms: CD97-targeted ADCs are currently supported by in vitro proof-of-concept evidence, whereas CAR-based approaches have preclinical in vivo evidence from animal models. RNA-mediated downregulation remains at an early proof-of-concept/target-validation stage and should not be considered an established CD97-specific RNA therapy. Strategies involving extracellular interaction blockade, 7TMD modulation, and pathway inhibition remain exploratory. Therapeutic feasibility also depends on surface antigen density and accessibility, cellular origin, normal-tissue expression and on-target/off-tumor risk, as well as delivery constraints such as the BBB/BTB in CNS tumors. Colors are used solely to distinguish the different therapeutic and translational strategy categories and do not represent their respective levels of evidence, whereas dashed borders indicate exploratory strategies that currently lack direct CD97-specific therapeutic validation. ADC, antibody–drug conjugate; BBB, blood–brain barrier; BTB, blood–tumor barrier; CAR, chimeric antigen receptor; CNS, central nervous system; DOPC, 1,2-dioleoyl-sn-glycero-3-phosphocholine; GAIN, GPCR autoproteolysis-inducing domain; GPS, GPCR proteolysis site; IHC, immunohistochemistry; LPAR1, lysophosphatidic acid receptor 1; miR-379-5p, microRNA-379-5p; mTORC2, mechanistic target of rapamycin complex 2; PI3K, phosphoinositide 3-kinase; THY1, Thy-1 cell-surface antigen; 7TMD, seven-transmembrane domain. Created in BioRender. Lei, Y. (2026) https://BioRender.com/j9ki5ys.

Current CD97-related therapeutic research focuses mainly on ADCs and CAR-based cellular therapies, but their levels of evidence differ: ADCs are currently supported by in vitro proof-of-concept evidence, whereas CAR-related strategies have demonstrated in vivo antitumor activity in animal models. RNA-mediated CD97 downregulation remains at an early exploratory stage, and structure-guided conformational modulation and signaling intervention remain primarily mechanistic or conceptual. Therapeutic evidence must therefore be distinguished from target-validation findings across platforms. The current therapeutic and translational framework for targeting CD97 is summarized in Figure 4.

A published study reported in vitro proof of concept for a CD97-targeted ADC. Antibodies directed against the CD97 GAIN region undergo CD97-dependent internalization, and ADCs constructed from these antibodies kill AML and GBM cells and patient-derived GSCs in vitro while showing relatively low in vitro cytotoxicity toward PBMCs from healthy donors [9]. The corresponding anti-CD97 antibodies and ADCs have also been disclosed in a published U.S. patent application [88]. Publication of a patent application records claimed compositions of matter; it does not constitute granted patent rights or clinical development. However, in vivo efficacy, pharmacokinetic, and systemic-toxicity data for a CD97 ADC in animal tumor models are not yet available. Low toxicity in PBMCs does not exclude on-target/off-tumor effects on smooth muscle, epithelial cells, or other normal tissues; the therapeutic window must therefore be tested in more complete normal-tissue panels and in vivo models.

In contrast, CD97-targeted CAR strategies have supporting evidence from animal models. CD97-directed CAR-Th9 cells suppress GSC-derived GBM and prolong animal survival [14]. In AML, CD97-directed CAR-T cells also show in vivo antileukemic activity, but CD97 expression by the therapeutic T cells causes fratricide. CRISPR-Cas9-mediated CD97 knockout improves CAR-T cell expansion, persistence, and produces antileukemic effects [19]. These studies provide in vivo proof of concept for CD97 targeting while highlighting fratricide and potential on-target/off-tumor risks associated with CD97 expression on therapeutic T cells and normal hematopoietic cells.

Beyond the target requirements of each platform, anatomical site also affects feasibility, with GBM providing a representative example. Patient-derived studies indicate enrichment of CD97 in GBM and GSCs with relatively low expression in the normal brain [14,15], suggesting that the tumor–normal brain differential may be more favorable than in some peripheral tumors. This expression differential, however, does not solve the central nervous system (CNS) delivery problem. The blood–brain barrier (BBB) and blood–tumor barrier (BTB) restrict the entry of antibodies, ADCs, and systemically administered cellular therapies, and barrier integrity varies among GBM regions. CD97-targeted therapy for GBM must therefore consider CNS delivery efficiency, including improved systemic trans-barrier delivery or local administration by intracerebral, intracavitary, or intraventricular routes [89].

RNA-mediated CD97 downregulation remains at an earlier stage. Small interfering RNA (siRNA)- and short hairpin RNA (shRNA)-mediated knockdown has been used in vitro and in vivo to validate CD97 functions in tumor proliferation, invasion, apoptosis, and stemness, but these studies primarily constitute target validation. Adenosine-to-inosine (A-to-I)-edited miR-379-5p provides another proof of concept: the edited miRNA directly binds the 3′ untranslated region (3′-UTR) of *ADGRE5* messenger RNA (mRNA) and reduces CD97 expression, and 1,2-dioleoyl-sn-glycero-3-phosphocholine (DOPC) nanoliposomal delivery of the corresponding miRNA mimic suppresses tumor growth in breast and lung cancer xenograft models [90]. The miRNA was not designed specifically against CD97 and may act through multiple targets; it therefore should not be regarded as an established CD97-specific RNA therapy.

Beyond reducing CD97 expression or directly eliminating CD97-positive cells, structural work suggests new strategies for modulating receptor activity. Structures of inactive and active CD97, the TIA-binding pocket, and the G13-coupling interface provide a basis for developing conformation-selective modulators [11,12]. Recent de novo protein-design studies of GPCRs further demonstrate that receptor structures can guide the design of high-affinity miniproteins with agonist or antagonist activity and that functional modulators can be identified by high-throughput screening; this strategy has been validated across several GPCRs but has not yet been applied directly to CD97 [91]. Together with the resolved CD97 7TMD conformations and TIA pocket, these advances provide a structural rationale for exploratory structure-guided modulation of CD97. Links between CD97 and LPAR1 [37,44], IL-8/CXCR2-PI3K/AKT, mTORC2-AKT, and MAPK pathways also suggest opportunities for signaling blockade or combination therapy [14,15,16,57]. These strategies nevertheless remain mainly mechanistic or conceptual, with no direct in vivo evidence for CD97-selective conformational modulators.

Overall, evidence for CD97-targeted therapy remains uneven: CAR-related strategies have supporting animal-model data, ADCs have in vitro proof-of-concept support, RNA-mediated downregulation remains at an early stage of development, and conformational modulation or signaling blockade remains exploratory. Therapeutic feasibility depends on tumor functional dependence, tumor–normal antigen separation, receptor state and internalization, and platform-specific tissue exposure and delivery. The current evidence, representative models, and major challenges for these therapeutic strategies are summarized in Table 2.

## 7. Future Directions: From Mechanistic Understanding to Clinical Translation

Future CD97 research must move beyond expression associations toward functional dependence. Existing studies show pronounced variation in CD97 expression, subcellular localization, and signaling output among tumor types, but systematic comparisons are still needed to identify tumors that genuinely depend on CD97 for growth, invasion, or survival. Cell-type-specific genetic manipulation, spatial analysis, and immunocompetent models should help distinguish tumor-cell-autonomous effects, microenvironmental effects, and normal-tissue functions and enable more accurate assessment of both the antitumor benefits and physiological costs of CD97 intervention.

Patient stratification likewise cannot rely only on total RNA expression or tissue positivity. More informative platform-specific variables include the cellular source of CD97, cell-surface antigen density, epitope accessibility, receptor processing and internalization, and expression differences between tumors and critical normal tissues. Multiplex immunohistochemistry, flow cytometry, single-cell analysis, and spatial multi-omics can provide complementary information. For CNS tumors such as GBM, target expression must also be evaluated together with BBB/BTB status, regional tumor heterogeneity, and conditions for local delivery.

Different CD97-targeted strategies also impose different requirements on the target. ADCs require sufficient surface antigen and efficient internalization; CAR-related therapies are more vulnerable to normal-tissue expression, potential on-target/off-tumor toxicity, and tissue-homing constraints; and conformational or signaling interventions require identification of receptor states and signaling nodes that are pharmacologically tractable. The priority is therefore not simply to add new intervention formats, but to determine which CD97 state is best matched to which therapeutic platform in each tumor context.

Overall, the clinical potential of CD97 will depend on the true degree of tumor dependence, effective platform exposure within the tumor, and the safety margin permitted by normal tissues. Building direct functional and translational evidence around these questions will be essential to move CD97 research from proof of concept toward clinical application.

## 8. Conclusions

CD97/*ADGRE5* is an aGPCR that integrates adhesion, mechanical stimulation, and GPCR signaling, and its cancer-associated functions depend on receptor state, cellular context, and the local microenvironment. Current evidence supports roles for CD97 in stemness maintenance, invasion and metastasis, metabolic adaptation, and stress survival in selected tumors, although the strength of evidence varies among mechanisms. Therapeutically, CAR-related strategies have shown preclinical antitumor activity in animal models, CD97-targeted ADCs are currently supported by in vitro proof-of-concept evidence, and RNA-mediated downregulation and structure-guided conformational modulation remain early exploratory directions. Because CD97 is also expressed by normal immune cells, smooth muscle, and selected epithelial and other normal tissues, clinical translation will require establishing tumor functional dependence together with sufficient tumor–normal antigen separation, careful assessment of potential on-target/off-tumor toxicity, and a platform-specific therapeutic window.

**Data Sources and Analytical Methods**

Publicly available bulk and single-cell RNA-sequencing data were used for the exploratory analyses presented in Figure 3. For Figure 3A, sample-level *ADGRE5* expression values from The Cancer Genome Atlas were obtained through the Human Protein Atlas and are reported as protein-coding transcripts per million. For Figure 3B–D, single-cell RNA-sequencing datasets were obtained from the Gene Expression Omnibus, ArrayExpress, and OMIX repositories. Dataset accessions, disease contexts, study designs, and corresponding references are provided in Table S1. Original author-provided cell annotations were retained where available and harmonized into major epithelial, immune, and stromal lineages. Epithelial cells from normal tissues were classified as normal epithelial cells. Tumor epithelial cells were classified as malignant epithelial cells when supported by explicit author annotations or patient-level CopyKAT inference using matched normal cells as the diploid reference. Figure 3B includes 13 solid-tumor cohorts containing patient-matched normal and tumor tissues. Figure 3C includes three normal brain samples, five high-grade gliomas, and thirteen pilocytic astrocytomas from GSE249263. Figure 3D includes five healthy bone-marrow donors and twelve patients with newly diagnosed acute myeloid leukemia from GSE116256. The comparisons in Figure 3C,D were unpaired.

Cells with non-zero *ADGRE5* expression were considered *ADGRE5*-positive. The detection fraction was calculated as 100 times the number of *ADGRE5*-positive cells divided by the total number of cells in the corresponding group. In Figure 3B, bubble size represents the *ADGRE5*-positive cell fraction for each dataset, condition, and lineage. In Figure 3C,D, bubble size represents the median sample-level or donor-level detection fraction, respectively. Bubble color represents the within-study percentile rank of median *ADGRE5* expression at the patient, sample, or donor level. Colors can therefore be compared among conditions and cell lineages within the same dataset but do not represent directly comparable absolute expression levels across datasets. Each dataset was analyzed separately, and the results were interpreted descriptively. Plot-level values and the underlying patient-, sample-, and donor-level data are provided in Data S2.

Single-cell RNA-sequencing data were processed using R version 4.5.1 and Seurat version 5.4.0. Copy-number variation inference was performed using CopyKAT version 1.1.0. Final figures were generated using R version 4.3.1 and ggplot2 version 3.4.2.

During the preparation of this manuscript, ChatGPT (GPT-5.5; OpenAI; https://chatgpt.com/) was used solely for language polishing and improving readability. The tool was not used for generating scientific content, literature interpretation, data analysis, figure generation, study design, or formulation of conclusions. All AI-assisted edits were reviewed and revised by the authors, who take full responsibility for the final content of the manuscript.

## Figures and Tables

**Table 1:** Structural determinants of CD97 activation, function, and therapeutic targetability.

Structural Feature	Principal Molecular Characteristics	Functional Significance	Targeting Implications	References
Extracellular EGF-like domains and splice isoforms	Variable numbers of EGF-like repeats generated by alternative splicing, usually comprising three, four, or five EGF-like domains	Provide the main ligand-binding platform and influence recognition of extracellular ligands such as CD55 and glycosaminoglycans (GAGs)	Determine antibody-epitope selection and ligand-blocking strategies and influence antibody recognition across isoforms and cellular contexts	[20,21,22,23,24]
GAIN/GPS module	GAIN domain containing the GPS autoproteolysis site	Controls receptor processing, formation of the noncovalent NTF–CTF complex, and accessibility of the TIA/Stachel sequence	Influences receptor maturation, cleavage state, and extracellular epitope exposure; an important region for GAIN/ENT-targeted antibodies and ADC design	[9,26,31]
TIA/Stachel tethered agonist sequence	Tethered/intramolecular agonist sequence at the GAIN-7TMD activation interface	Interacts with the 7TMD core when accessible, promoting receptor activation and GPCR signaling	Provides a mechanistic rationale for potential activation-state-selective targeting and signaling blockade	[11,12,31,35]
7TMD core and G-protein-coupling interface	Seven-transmembrane domain with a preferential G13-coupling interface	Mediates G-protein coupling and contributes to receptor conformational states that determine downstream signaling	Exploratory target region for conformation-locking small molecules or allosteric modulators	[11,12,36]
Intracellular C-terminal tail and PDZ-binding motif	Cytoplasmic tail containing phosphorylation sites and a C-terminal PDZ-binding motif	Regulates β-arrestin recruitment, PDZ-scaffold interactions, cytoskeletal organization, and force-dependent cell detachment	Highlights intracellular regulatory mechanisms that may alter CD97 signaling and cell behavior	[15,34]
Glycosylation and epitope accessibility	N-glycosylation within EGF-like domains	Regulates ligand binding, antibody recognition, and epitope exposure	May affect antibody-based target recognition, interpretation of CD97 surface positivity, and selection of detection reagents	[27]

**Table 2:** Platform-specific evidence and major challenges for CD97-targeted therapeutic strategies.

Strategy	Mechanistic Rationale	Representative Models	Key Evidence	Current Evidence Level	Major Challenges	References
ADC	Delivery of cytotoxic payloads through internalizing extracellular epitopes	AML, GBM, and patient-derived GSCs	GAIN-targeted ADCs show CD97-dependent internalization and in vitro cytotoxicity in AML, GBM, and patient-derived GSCs; limited in vitro toxicity toward healthy-donor PBMCs does not establish systemic safety	In vitro proof-of-concept	Epitope specificity, internalization, antigen heterogeneity, normal-tissue expression, and potential on-target/off-tumor toxicity	[9,92]
CAR-T/CAR-Th9	Direct recognition and elimination of CD97-expressing tumor cells	GBM/GSC and AML models	CD97-directed CAR strategies show antitumor activity in GBM and AML animal models; CD97 knockout reduces CAR-T cell fratricide	Preclinical in vivo evidence	Antigen heterogeneity, fratricide, solid-tumor homing, normal-tissue expression, and on-target/off-tumor toxicity	[14,19,93]
RNA-mediated CD97 downregulation	Reduction in *ADGRE5*/CD97 expression by an edited miRNA rather than direct elimination of CD97-positive cells	Breast and lung cancer xenograft models	Edited miR-379-5p directly targets *ADGRE5*, reduces CD97 expression, and suppresses xenograft growth, but it is not a CD97-specific therapeutic molecule	Early proof-of-concept/target-validation evidence	RNA stability, tumor-selective delivery, off-target effects, endosomal escape, and immunogenicity	[90]
7TMD conformational modulation	Modulation of TIA/Stachel–7TMD–G-protein signaling without cell depletion	Cryo-EM structural studies	CD97 structures define active and inactive conformations and reveal potentially targetable TIA-binding and G-protein-coupling interfaces	Exploratory/structural rationale	Lack of selective compounds, receptor selectivity, and pharmacological validation	[11,12]
Combined pathway inhibition	Targeting CD97-associated stemness, EMT, and metabolic signaling	GBM/GSC and inflammation-driven tumor models	CD97 is linked to mTORC2-AKT, MAPK, and IL-8/CXCR2-PI3K/AKT signaling in tumor models	Exploratory/mechanistic rationale	Pathway redundancy, systemic toxicity, target specificity, and patient selection	[14,15,16,57]

## Data Availability

No new data were generated in this study. Publicly available datasets were analyzed as described below. The bulk RNA-expression data shown in Figure 3A were obtained from The Cancer Genome Atlas through the Human Protein Atlas (https://www.proteinatlas.org/ENSG00000123146-ADGRE5/cancer; accessed on 16 June 2026) and are provided as Data S1. The single-cell RNA-sequencing analyses shown in Figure 3B–D used publicly available datasets deposited in the Gene Expression Omnibus, ArrayExpress, and OMIX repositories. The dataset accession numbers, disease contexts, comparators, and corresponding references are listed in Table S1. Processed cohort metadata, plot-level values, and patient-, sample-, and donor-level source data underlying Figure 3B–D are provided as Data S2. All other information discussed in this review is available in the cited publications.

## Supplementary Materials

The following supporting information can be downloaded at https://www.mdpi.com/article/10.3390/cells15171605/s1. Table S1: Summary of the single-cell RNA-sequencing datasets used in Figure 3B–D; Data S1: Human Protein Atlas/TCGA bulk *ADGRE5* RNA-expression data underlying Figure 3A; Data S2: Processed single-cell *ADGRE5* source data, cohort metadata, and data dictionary underlying Figure 3B–D.

## Abbreviations

The following abbreviations are used in this manuscript:

3′-UTR	3′ untranslated region
7TMD	seven-transmembrane domain
ADC	antibody–drug conjugate
ADGRE5	adhesion G-protein-coupled receptor E5
aGPCR	adhesion G-protein-coupled receptor
AI	artificial intelligence
AKT	protein kinase B
AML	acute myeloid leukemia
A-to-I	adenosine-to-inosine
BBB	blood–brain barrier
BTB	blood–tumor barrier
CALR	calreticulin
CAR	chimeric antigen receptor
CAR-T	chimeric antigen receptor T cell
CAR-Th9	chimeric antigen receptor-engineered T helper type 9 cell
cDC2	conventional type 2 dendritic cell
CNS	central nervous system
CopyKAT	Copy Number Karyotyping of Aneuploid Tumors
CRISPR-Cas9	clustered regularly interspaced short palindromic repeats-CRISPR-associated protein 9
CTC	circulating tumor cell
CTF	C-terminal fragment
CXCR2	C-X-C motif chemokine receptor 2
DC	dendritic cell
DLG1	discs large homolog 1
DOPC	1,2-dioleoyl-sn-glycero-3-phosphocholine
ECL1/ECL2	extracellular loops 1 and 2
EGF	epidermal growth factor
EGF-TM7	epidermal growth factor-seven-transmembrane
EMT	epithelial–mesenchymal transition
ENT	extracellular N terminus
ERK	extracellular signal-regulated kinase
Fc	fragment crystallizable
FLT3-ITD	FMS-like tyrosine kinase 3 internal tandem duplication
G13	heterotrimeric G-protein G13
GAG	glycosaminoglycan
GAIN	GPCR autoproteolysis-inducing domain
GBM	glioblastoma
GEO	Gene Expression Omnibus
GLUT1	glucose transporter 1
GMP	granulocyte–monocyte progenitor
GPCR	G-protein-coupled receptor
GPS	GPCR proteolysis site
Gq	heterotrimeric G-protein Gq
Gs	heterotrimeric G-protein Gs
GSC	glioblastoma stem-like cell
HGG	high-grade glioma
HSC	hematopoietic stem cell
HSPC	hematopoietic stem/progenitor cell
HT1080	human fibrosarcoma cell line
ICT	intracellular C terminus
IHC	immunohistochemistry
IL-8	interleukin-8
IUPHAR	International Union of Basic and Clinical Pharmacology
JAK2	Janus kinase 2
Kgp	lysine-specific gingipain
LDHA	lactate dehydrogenase A
LPA	lysophosphatidic acid
LPAR1	lysophosphatidic acid receptor 1
LSC	leukemia stem cell
M0	unpolarized macrophage
MAPK	mitogen-activated protein kinase
miR-379-5p	microRNA-379-5p
miRNA	microRNA
MMP-9	matrix metalloproteinase-9
mRNA	messenger RNA
mTORC2	mechanistic target of rapamycin complex 2
NK	natural killer
NTF	N-terminal fragment
OMIX	Open Archive for Miscellaneous Data
PA	pilocytic astrocytoma
PBM	PDZ-binding motif
PBMC	peripheral blood mononuclear cell
PDB	Protein Data Bank
PDZ	PSD-95/Dlg/ZO-1
PI3K	phosphoinositide 3-kinase
pTPM	protein-coding transcripts per million
RGD	arginine–glycine–aspartate
RNA	ribonucleic acid
RNA-seq	RNA sequencing
shRNA	short hairpin RNA
siRNA	small interfering RNA
ST6GAL1	β-galactoside α-2,6-sialyltransferase 1
STAT3	signal transducer and activator of transcription 3
TCGA	The Cancer Genome Atlas
THP-1	human monocytic leukemia cell line
THY1	Thy-1 cell-surface antigen
TIA	tethered/intramolecular agonist
TM6/TM7 (TMH6/TMH7)	transmembrane helices 6 and 7
