# Supplementary material for: CD97/ADGRE5 in Cancer: Structural Activation, Context-Dependent Signaling, and Therapeutic Targeting
Source: Cells. 2026 Sep 3;15(17):1605. doi: 10.3390/cells15171605 (PMC13565010; doi:10.3390/cells15171605)
Supplement: Supplementary file 1 [file cells-15-01605-s001.zip › Table_S1_Single_Cell_RNA_Seq_Datasets_Figure_3B-D.pdf]

Supplemental Table S1. Summary of Single-Cell RNA-Seq Datasets Used in Figure 3B-D.

Dataset	Disease Context / Comparator	Abbreviation	Reference PMID	Figure
GSE149614	Hepatocellular carcinoma	HCC	35933472	3B
GSE212966	Pancreatic ductal adenocarcinoma	PDAC	36944944	3B
GSE202813	Clear-cell renal cell carcinoma	ccRCC	38281962	3B
GSE132465	Colorectal cancer	CRC	32451460	3B
GSE206785	Gastric cancer	GC	36550535	3B
GSE184362	Papillary thyroid carcinoma	PTC	34663816	3B
GSE138709	Intrahepatic cholangiocarcinoma	iCCA	32505533	3B
GSE131907	Lung adenocarcinoma	LUAD	32385277	3B
GSE181294	Prostate adenocarcinoma	PRAD	36750562	3B
GSE196756	Esophageal squamous cell carcinoma	ESCC	N/A*	3B
GSE178481	Clear-cell renal cell carcinoma	ccRCC	36180422	3B
OMIX001111	Breast cancer	BC	36040519	3B
E-MTAB-11948	Cervical cancer	CC	36357663	3B
GSE249263	Normal brain, high-grade glioma and pilocytic astrocytoma	HGG/PA	39137048	3C
GSE116256	Newly diagnosed AML and healthy donor bone marrow	AML	30827681	3D

* GSE196756 has no publication linked in the GEO record; therefore, no PMID is available. **Abbreviations:** AML, acute myeloid leukemia; BC, breast cancer; CC, cervical cancer; ccRCC, clear-cell renal cell carcinoma; CRC, colorectal cancer; ESCC, esophageal squamous cell carcinoma; GC, gastric cancer; HCC, hepatocellular carcinoma; HGG, high-grade glioma; iCCA, intrahepatic cholangiocarcinoma; LUAD, lung adenocarcinoma; PA, pilocytic astrocytoma; PDAC, pancreatic ductal adenocarcinoma; PRAD, prostate adenocarcinoma; PTC, papillary thyroid carcinoma.
